# Neuroglobin Over Expressing Mice: Expression Pattern and Effect on Brain Ischemic Infarct Size

**DOI:** 10.1371/journal.pone.0076565

**Published:** 2013-10-01

**Authors:** Zindy Raida, Christian Ansgar Hundahl, Jens R. Nyengaard, Anders Hay-Schmidt

**Affiliations:** 1 Department of Neuroscience and Pharmacology, University of Copenhagen, Copenhagen, Denmark; 2 Centre of Excellence for Translational Medicine, University of Tartu, Tartu, Estonia; 3 Stereology and Electron Microscopy Laboratory, Centre for Stochastic Geometry and Advanced Bioimaging, Aarhus University, Aarhus, Denmark; Biological Research Centre of the Hungarian Academy of Sciences, Hungary

## Abstract

**Background:**

Stroke is a major cause of death and severe disability, but effective treatments are limited. Neuroglobin, a neuronal heme-globin, has been advocated as a novel pharmacological target in combating stroke and neurodegenerative disorders based on cytoprotective properties. Using thoroughly validated antibodies and oligos, we give a detailed brain anatomical characterization of transgenic mice over expressing Neuroglobin. Moreover, using permanent middle artery occlusion the effect of elevated levels of Neuroglobin on ischemic damage was studied. Lastly, the impact of mouse strain genetic background on ischemic damage was investigated.

**Principal Findings:**

A four to five fold increase in Neuroglobin mRNA and protein expression was seen in the brain of transgenic mice. A β-actin promoter was used to drive Neuroglobin over expression, but immunohistochemistry and *in situ* hybridization showed over expression to be confined to primarily the cortex, hippocampus, cerebellum, and only in neurons. The level and expression pattern of endogenous Neuroglobin was unaffected by insertion of the over expressing Ngb transgene. Neuroglobin over expression resulted in a significant reduction in infarct volume 24 hours after ischemia. Immunohistochemistry showed no selective sparing of Neuroglobin expressing cells in the ischemic core or penumbra. A significant difference in infarct volume was found between mice of the same strain, but from different colonies.

**Significance:**

In contrast to some previous reports, Neuroglobin over expression is not global but confined to a few well-defined brain regions, and only in neurons. This study confirms previous reports showing a correlation between reduced infarct volume and elevated Neuroglobin levels, but underlines the need to study the likely contribution from compensatory mechanisms to the phenotype following a genetic perturbation. We also stress, that care should be taken when comparing results where different mouse strains and colonies have been used due to large genetic background contribution to the observed phenotype.

## Introduction

Stroke is a major cause of death and severe disability due to an increasing number of individuals being affected by stroke each year [1], [2]. Stroke is the third most common cause of death in developed countries [3], and an enormous economic as well as social burden on society [4]. About 85% of all strokes are ischemic, and most often due to a blood clot blockage of a cerebral artery [5]. Despite intensive research in the field of ischemia, trombolysis within the narrow timeframe of three to six hours is to date the only effective treatment available [6], [7]. Once the cerebral artery has been occluded no clinical treatment can save brain cells and prevent irreversible damage. Therefore, there is an increasing interest in using potentially neuroprotective endogenous expressed proteins as targets for pharmacological stroke treatment, Neuroglobin (Ngb) being one of them. Ngb is advocated as a novel pharmacological target in combating stroke and neurodegenerative disorders, based on cytoprotective properties that has only been characterized to a limited extent [8]. Ngb was discovered in 2000 by Burmester and colleagues [9], who found Ngb to be expressed primarily in neurons of the mouse brain, hence the name Neuroglobin. Ngb is a heme-globin with an oxygen (O_2_) affinity in the range of that of myoglobin. Suggested functions of Ngb in the brain are storage and/or transportation of O_2_, [9]–[12], but also sensing and scavenging of O_2_, nitric oxide (NO), reactive oxygen species (ROS) [13]–[16] and cytochrome c (reviewed in [17]). Because of the aforementioned properties, Ngb is believed to play a neuroprotective role in conditions like hypoxia and ischemia, characterized by an imbalance in the oxygen metabolizing systems. This notion has gained further support due to the very high metabolic demand of the brain [18]. Although 13 years have passed since the discovery of Ngb, no underlying molecular mechanism or functional role has been conclusively identified. Numerous studies have shown that over expressing the levels of Ngb reduce infarct size [19]–[21], ROS and free radical formation [14], [15], [22] following ischemia. Also an increase in the Ngb immunoreactivity (IR) of the ischemic hemisphere, compared to the non-ischemic hemisphere, has been reported following transient occlusion of the middle cerebral artery (tMCAo) in wild type (WT) mice [23]. Contradictory to this one study, other studies have not been able to show any up regulation of Ngb following tMCAo [24] or global ischemia [25]. Using Ngb deficient mice (Ngb-null mice), we recently found the infarct volume to be significantly reduced, compared to WT controls 24 hours, after permanent middle cerebral artery occlusion (pMCAo) - thereby questioning the neuroprotective effects of Ngb at endogenous levels [26]. Moreover, a thorough characterization of two commonly used commercial Ngb antibodies showed ubiquitous staining when applied on brain slides from Ngb-null mice, and must be considered unreliable for immunohistochemical detection of Ngb expression in the mouse brain. The staining pattern of our own in house made antibodies were also validated on brain slides from Ngb-null mice were they produced no staining. Moreover, their specificity have been confirmed by ISH investigations [26], Western blot analysis [26], [27], pre-adsorption tests [28], [29] and finally the expression pattern match previous reports by Mammen et al [30] and the Allen Brain Atlas (www.brain-map.org). In light of recent conflicting results concerning the *in vivo* neuroprotective effect of Ngb and the specificity of various Ngb antibodies, the aims of this study were to re-investigate the location and type of the over expressing cells using our own in house made antibodies, as well as re-investigate the effect of Ngb over expression on brain infarct size. The Ngb over expressing (Ngb-Tg) mouse line, commercially available at JAX© Mice was characterized using Real Time Quantitative PCR (RT-QPCR), and Western blotting (WB) to quantify the level of over expression. Immunohistochemistry (IHC) and *In Situ* Hybridization (ISH) were used for anatomical characterization of Ngb protein and mRNA expression, and IHC was used to investigate whether Ngb is localized in astroglia as previously described [26]. Lastly, the effect of genetic background on the infarct volume was investigated by comparing the infarct volumes from one C57BL6 strain with the infarct volume produced in a different inbred colony originated from the same C57BL6 strain. This scenario is often the case when comparing results in different studies. We hereby provide a clearer picture of intra-strain variation, and how comparable the various studies using Ngb-Tg mice really are. We used the pMCAo model to induce ischemia and the infarct volumes were estimated using design based stereology. This is, to our knowledge, the first study to give a detailed anatomical characterization of Ngb expression in the Ngb-Tg mouse. In addition, this report is the first to measure the comparability of ischemic volumes between studies using different Ngb transgenic mouse lines founded on the same strain.

## Materials and Methods

The animal care and all experimental procedures performed in this study were approved in accordance with the European Community Counciĺs Directive of Nov 24^th^ 1986 (86/609/EEC) by The Danish National Committee for Ethics in Animal Research under the Danish Ministry of Justice (License number 2010/561-1834). All animals were housed in a fully AAALAC accredited facility, and procedures were performed in accordance with the *Guide for the care and use of Laboratory Animals* [31]. The animals were housed in a 12∶12 hour dark/artificial light cycle with lighting period starting at 6 a.m. Room temperature was maintained at 20±2°C, and air humidity was kept within the range of 30–60%. Authorized personnel carried out daily routines between 7 a.m. and 4 p.m. Standard laboratory food pellets and water was provided *ad libitum*. Post-operative, the animals received additional nutritional gel as well as soaked food pellets.

### Study design

A total of 47 animals were used for the experimental work. 18 male C57BL/6 mice, aged 10–14 weeks, were randomized to one of the following groups: I. WT pMCAo hsa1-background (n = 4), II. WT pMCAo Tg-background (n = 5), III. Ngb-Tg pMCAo (n = 9). Brains from the animals in all three groups were paraffin embedded, and sectioned for infarct volume estimations. No sham group was included, since our previous study showed no brain damage in sham operated animals [26]. All animals were euthanized after 24 hours. Ngb-Tg mice were compared with WT Tg-background littermates to investigate the influence of background variation on the infarct volume. Series from 3 Ngb-Tg animals from group number III and 3 native Ngb-Tg brains were used for IHC staining on paraffin sections to investigate whether Ngb is up-regulated in the penumbra zone following an ischemic injury.

26 naive male and female C57BL/6 mice, aged 8–16 weeks were randomized to one of the following groups: IV. WT native (n = 6) and V. Ngb-Tg native (n = 6) brains were perfusion fixed, and frozen sectioned for IHC and ISH investigations as described below. VI. WT native (n = 8), VII. Ngb-Tg (n = 4) and VIII. Ngb-null mice (n = 2) brains were homogenized and used for RT-QPCR. 3 WT, 3 Ngb-Tg and 1 Ngb-null mice from groups number VI, VII, and VIII were used for WB quantifications. These 26 animals were used to investigate the expression pattern of Ngb in the Ngb-transgenic mouse brain.

### Over expressing mouse model

The Ngb over expressing mouse line (B6.Cg-Tg(CAG-Ngb,-EGFP)1Dgrn/J) was bought from JAX© Mice (The Jackson Laboratory, Maine, USA). The Ngb-Tg mouse line was originally deposited by Dr. Greenberg, Buck Institute of Aging, CA, USA. The Ngb-Tg mice were made as described in [19], [32]. In brief, full-length murine *Ngb* cDNA was subcloned into a GFP containing pTR-UF12d vector, and expression of Ngb and GFP was driven by a chicken β-actin promoter with a CMV enhancer. BDF x CD1 mice were used as founders. At the Jackson laboratory, Ngb-Tg mice were backcrossed to C57BL/6J for at least five generations to generate the congenic strain used in this study.

### Genotyping PCR

Mouse tail DNA extraction and PCR was performed to confirm the genotypes as described in [27], using the following primer set in 0.2 µM concentration. GFP: forward 5′-GCGGTCACAAACTCCAGCAGGACCA-3′ and reverse 5′-GGCGTGGTCCCAATCTCGTGGAA-3′giving a 664-bp product. No band yielded WT mice and one band for the presence of GFP yielded Tg mice.

### Perfusion fixation and tissue handling

For IHC and ISH six male WT and six male Ngb-Tg mice were perfusion fixed with phosphate buffered 10% formalin. Brains were removed and post-fixed in same fixative for 24 hours at 4°C, followed by cryoprotection in 30% sucrose/PBS solution for five days, and stored at –80°C until use. Frozen brains were cut in 40 µm coronal sections in series of four, and stored at 4°C in PBS containing 0.1% NaAzid.

Brains used for volume estimation and IHC on paraffin sections were perfusion fixed as previously described [33], before they were embedded in paraffin, and cut into 40 µm thick coronal slices in series of 4 on a MICROM HM 355 (Brock and Michelsen A/S, Denmark). The section distance between the slices in one sample series were 600 µm. Sectioning and staining with Mayeŕs haematoxylin (Mayer from Th. Geyer Denmark Aps) was also performed, as previously described [26].

### RT-PCR and Real Time Quantitative PCR

Eight 8 weeks old male WT, two Ngb-null mice and four Ngb-Tg mice were used. Total brain RNA was extracted using the PARIS Kit (Life Technologies, Cat# AM1921), as described in [34]. cDNA was made from 1 µg RNA using random hexamers primers and SuperScript III Reverse Transcriptase (Life Technologies, Cat# 18080-044), according to manufacture guidelines. For RT-QPCR cDNA was diluted 1∶1 in replicas of four and expression of the Ngb transcript was quantified, using a Taqman gene expression assay (Life Technologies, Mm00452101_m1). Ngb expression was normalized to Hypoxanthine phosphoribosyltransferase 1 (Hprt1): Mm01545399_m1, using the delta-delta-CT method [35]. cDNA from two Ngb-null mice were used to test the specificity of the Ngb primers. No amplification was detected.

### Electrophoreses and Western blotting

For WB, brains were removed from three male WT, three Ngb-Tg mice and one Ngb-null mouse, and divided into the two hemispheres, snap frozen on dry ice, and stored at –80°C until protein extraction.

Protein extraction, electrophoreses and protein transfer was performed as described in [34]. Electrophoreses was performed using a 4–12% Bis-Tris gel (Life Technologies, Cat# NP0321PK2). For WB the membrane was blocked in PBS containing 3% nonfat dry milk for 1 hour at RT. After blocking, the membrane was transferred to antibody incubation buffer (PBS-0.1% Tween-20 and 3% nonfat dry milk), containing guinea pig anti-Ngb (in house made, code G in 1∶500 dilution), and rabbit anti-β-actin as loading control (Cell Signaling, Cat# 4970, in 1∶15000 dilution) for 1 hour at RT, followed by overnight incubation at 4°C on a rocking table. Antibodies were washed off by 6x MilliQ water steps. The primary antibodies were detected with donkey anti-guinea pig Alexa-680 and donkey anti-rabbit Alexa-790, conjugated secondary antibodies (Jackson ImmunoResearch, Cat #706-625-148 and 711-655-152 both in 1∶40000 dilutions) in PBS-0.1% Tween-20 for 1 hour at RT. The secondary antibodies were washed off by 6x MilliQ water steps, followed by a 30 minutes wash in PBS-0.1% Tween-20. The membrane was washed 6x in MilliQ water just before signal detection. For detection we used the Li-Cor Odyssey CLx system (Li-Cor Biosystems-GmbH, Germany). Images were gray scaled, and quantification was performed according to the NIH guidelines, by use of the Gel-analyzer plugin in ImageJ®.

### Immunohistochemistry

Diaminobenzidine (DAB) IHC was performed using an in house made polyclonal rabbit anti-Ngb antibody (code #4836/5) as described in [26]. To distinguish the cell type origin of Ngb over expressing cells, double IHC was performed as described in [33] with Ngb (guinea pig anti-Ngb #G, dilution 1:500), neuronal marker NeuN (Millipore, Cat #MAB377B, dilution 1∶1000) and Glial Fibrillary Acidic Protein (GFAP) (DAKO, Cat #Z0334, dilution 1∶1000). Ngb was detected with a goat anti-guinea pig conjugated Alexa-488 (Invitrogen, Cat #A11073, dilution 1∶500), NeuN with a streptavidin conjugated Alexa-680 (Invitrogen, Cat #S21378, dilution 1∶500), and GFAP was detected with a goat anti-rabbit conjugated Alexa-594 (Invitrogen, Cat #A11072, dilution 1∶500). Images were acquired using a Zeiss LSM-780 confocal microscope with appropriate filter settings.

For paraffin IHC, sections were deparaffinized without antigen retrieval, and processed as described in [26]. After IHC, the sections were counterstained with Mayeŕs haematoxylin.

### *In situ* hybridization

Free-floating ISH was performed as described in [33].

### Surgical procedure

Anesthesia, as well as the monitoring of physiological parameters, was performed as described in [26], yet systolic blood pressure and heart rate was not measured. In the previous study, measurements were done non-invasively, giving some problems getting the transducer to measure on about 50% of the mice. This was due to differences in tail diameter and muscle mass around the tail vein. We therefore started to measure invasively instead. Invasive blood pressure measurements require catheterization of the femoral artery to gain arterial access. This operation prolonged our operation and anesthesia time significantly. Due to the known neuroprotective properties of Sevoflurane [36], we decided to leave out this measurement in order to keep the anesthesia time as low as possible. In our previous study [26] we did not observe any blood pressure measurements outside the normal physiological range, or any significant differences in heart rate between the groups. We believe this is also the case in this identical set-up. Physiological parameters measured are shown in Table 1.

**Table 1:** Physiological parameters.

Group	PMCAo WT (n = 9)	PMCAo Ngb-Tg (n = 9)
Mean pCO_2_ in the exhaust air (kPa)	38.62 ±0.89	36.63±0.90
Rectal Temp. (°C)	36.64±0.11	36.53±0.11
Body weight preoperative (gram)	32.51±0.69	30.58±0.62
Body weight, day of sacrifice (gram)	30.87±0.61	28.78±0.65
Anesthesia time	9.89±0.66	8.78±0.28

Physiological parameters monitored during the operational procedure both groups. Mean values ± SE. Mann-Whitney analysis was used for the comparison between the groups.

The surgical procedure and post-operative care was performed as described in [26]. In brief, an incision through the temporal muscle between the lateral part of the orbit and the external meatus was made. A hole was drilled directly over the distal part of the right MCA, dura mater was removed, and the MCA was electrosurgically coagulated.

### Stereology

For stereological counting we used an Olympus MVX10 MacroView microscope, equipped with an Olympus DP71 digital camera coupled to a PC for estimating infarct volume. Infarct volumes were estimated on heamatoxylin stained coronal brain slides, by use of Cavalieris estimator and a 2D nucleator and Cavalierís principle as described in [26]. The Cavalierís estimator states that the volume of any arbitrarily shaped object may be estimated by sectioning it into parallel planes with known section distance, and measuring the cross sectional area of interest in each plane [37]. Using NewCAST software (Visiopharm A/S, Hørsholm, Denmark), the center of the infarct was manually marked as the origin of the nucleator. The software then generates four systematic random directions used for area measurements. Manually on the computer screen, the demarcation between infarct borders and the lines were marked. The software then automatically estimates the infarct area on each slide. According to the Cavalieris estimator, the total volume was calculated by multiplying each area with the distance between every slide.

### Statistics

Power analysis showed that an effect of the treatment of approximately one standard deviation in the outcome could be obtained using five to seven mice, giving a power of 80% to detect a statistical significant difference. We observed a statistically significant difference of treatment, which suggests that the study was adequately powered. Statistical comparisons were made using Mann-Whitney two tailed tests, and p-values <0.05 were considered significant. All data was analyzed using GraphPad Prism software.

## Results

### Dropouts

One brain from a Ngb-Tg mouse was damaged during preparation. We therefore ended up with Ngb-Tg pMCAo (n = 8), and WT pMCAo (n = 9) for infarct volume estimations. There was one outlier in the Ngb-Tg group with an observation that fell outside of the mean by more than 2 SD. Therefore the final volume estimation result is based on brains from five WT mice with Tg-background, seven Ngb-Tg littermates and four WT mice with hsa1 background for detection of background differences.

### Level of over expression by RT-QPCR and WB (Figure 1)

**Figure 1:**
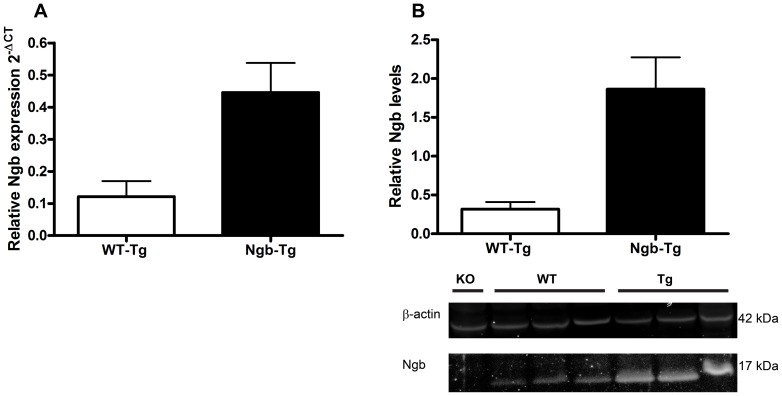
Genotyping and Western blot analysis of Ngb over expression. (**A**) RT-QPCR showed an approximately four fold increase in Ngb mRNA expression in Tg (n = 4, 0.446 SD±0.092) mice compared to WT (n = 8, 0.121 SD±0.048). (**B**) Western blot analysis confirmed the increase in mRNA showing an approximate five-fold increase in Ngb expression in Tg (n = 3, 1.864 SD±0.410) compared to WT (n = 3, 0.317 SD±0.091) mice. WB of ß-actin and Ngb are shown under the graph.

Tail genotyping PCR showed the expected 664-bp product corresponding to GFP in the Tg mice. RT-QPCR and WB analysis showed an approximate four to five-fold increase in Ngb mRNA and protein expression in the brains of Ngb-Tg mice, compared to WT littermates (Figure 1A-B).

### Overview of Ngb mRNA and Ngb-IR in brains of Ngb-Tg and WT mice (Figure 2)

**Figure 2:**
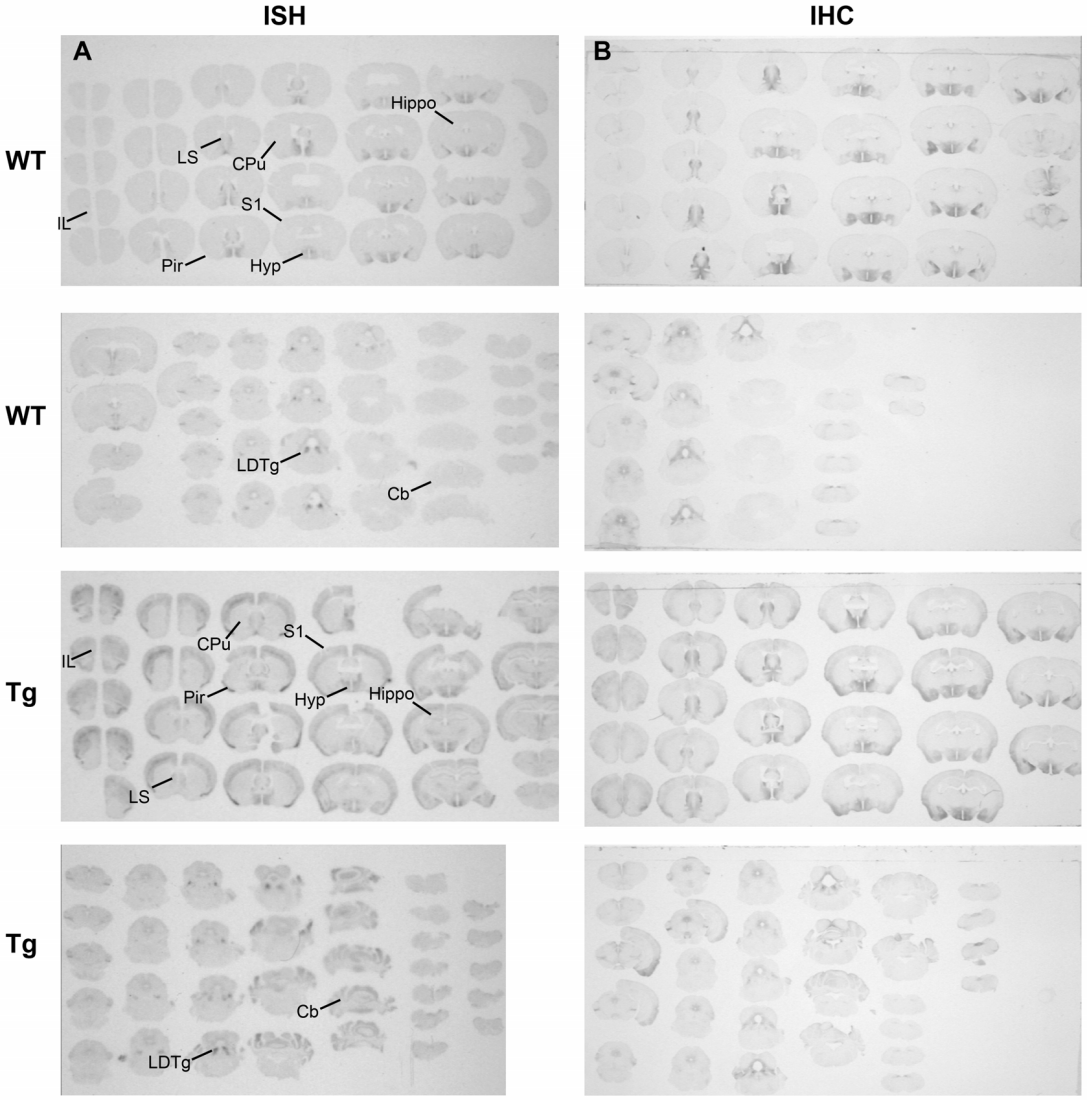
Overview of Ngb mRNA and protein expression in WT and Ngb-Tg brain. (**A**) In Situ Hybridization (ISH) and (**B**) immunohistochemistry (IHC) showed distinct and pronounced Ngb expression in hypothalamus and tegmental areas of the brain of both WT and Tg mice. Differential Ngb expression was observed primarily in the cortical areas, hippocampus caudate putamen and cerebellum of Tg mice. Abbreviations: Caudate putamen (CPu); Cerebellum (Cb); Hypothalamus (Hyp); Hippocampus (Hippo); Infralimbic area (Il); Laterodorsal tegmental nucleus (LDTg); Lateral septum (LS); Piriform cortex (Pir); Primary somatosensory cortex (S1).

A perfect overlap was observed between the Ngb mRNA (Figure 2A) and IR (Figure 2B) in both WT and Ngb-Tg mice. The most pronounced differences in Ngb mRNA, and IR between WT and Ngb-Tg mice, were seen in the cortical areas, hippocampus, caudate putamen (CPu) and cerebellum. Endogenous Ngb expression in Ngb-Tg mice was comparable both in intensity and localization to that of WT mice.

### Immunohistochemical characterization of Ngb-IR in Ngb-Tg mice (Figures 3, 4, 5, 6)

**Figure 3:**
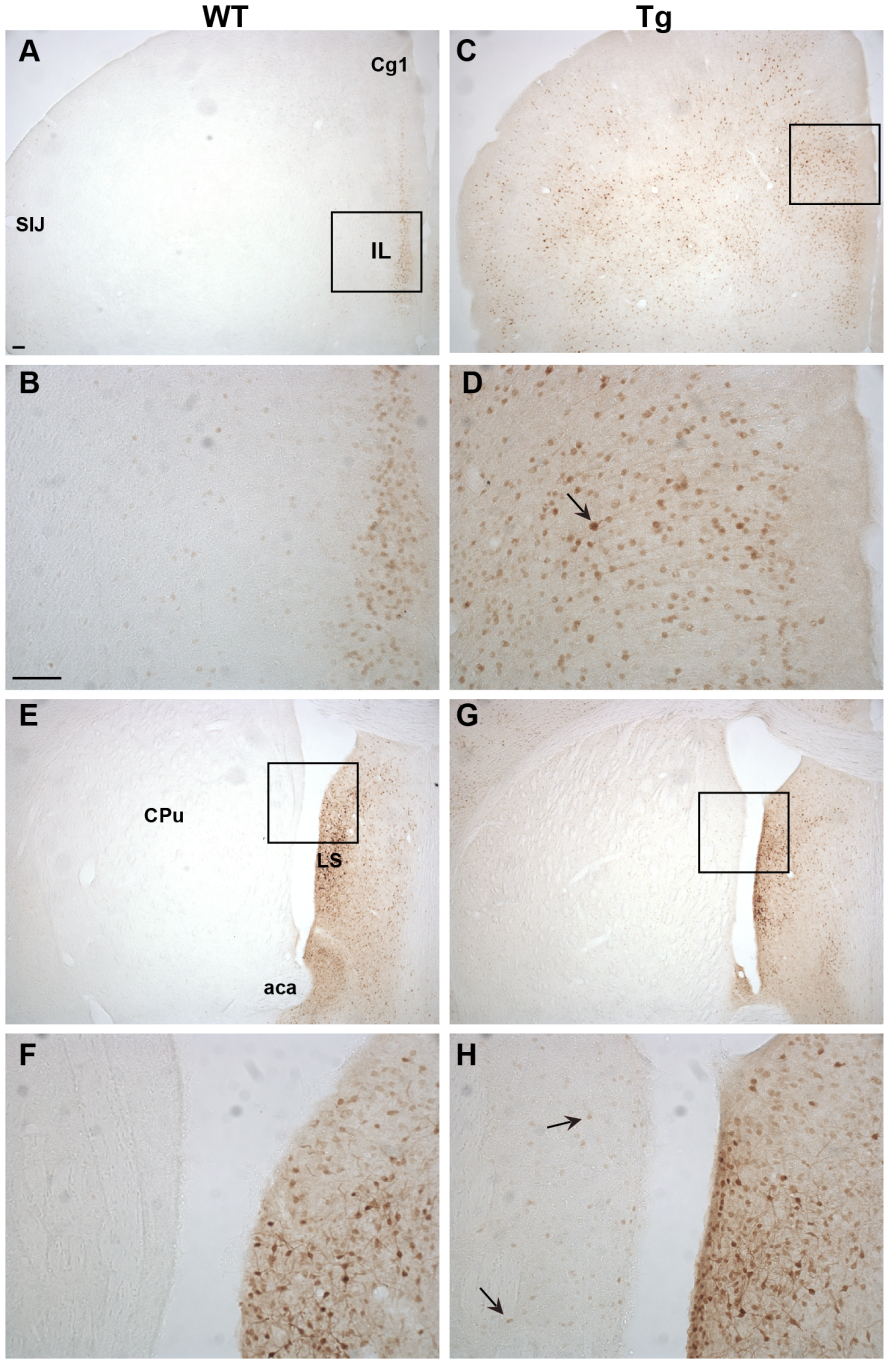
Ngb immunoreactivity (IR) in the forebrain. WT Ngb-IR was mostly confined to a thin layer of the Il and Cg1 (**A-B**). In Tg mice Ngb-IR was seen throughout the forebrain in medium intense stained cells (**C-D**). Intense Ngb-IR could be seen in cell bodies and processes in LS of WT (**E-F**) and Tg mice (**G-H**). In Tg mice weak Ngb-IR could also be seen scattered throughout the CPu in small sized cells. Black arrow exemplifies an over expressing cell. Abbreviations: Anterior commissure, anterior part (aca); Cingulate cortex, area 1 (Cg1); Infralimbic area (Il); Lateral septum (LS); Primary somatosensory cortex, jaw region (SIJ). Scale bar 100 µm.

**Figure 4:**
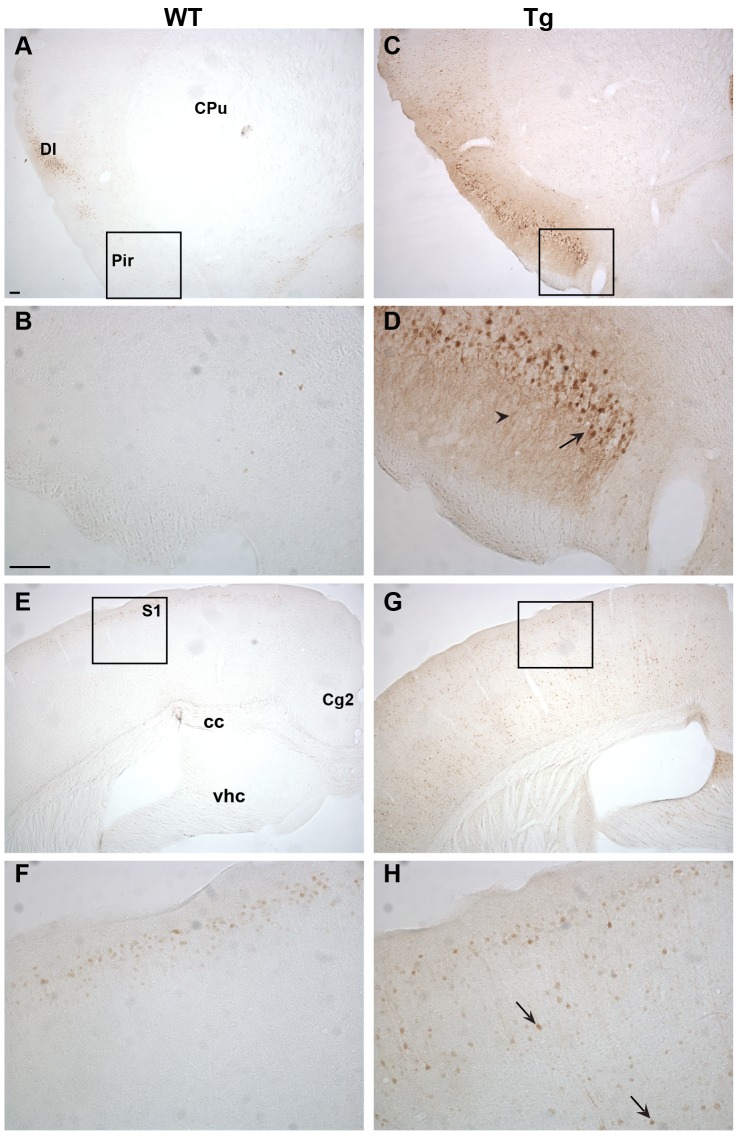
Ngb-IR in cortical areas. In the piriform cortex Ngb-IR was only seen in few cells of WT mice (**A-B**). Conversely, intense Ngb-IR was seen in cell bodies and processes throughout the piriform cortex of Tg mice (**C-D**). In WT mice Ngb-IR was confined to 2–3 cell layers of the S1 (**E-F**) whereas in Tg mice Ngb-IR was seen scatter throughout all cortical layers (**G-H**). Black arrow exemplifies an Ngb over expressing cell body and black arrowhead over expressing processes. Abbreviations: Caudate putamen (CPu); Cingulate cortex, area 2 (Cg2); Corpus callosum (cc); Dysgranular insular cortex (DI); Piriform cortex (Pir); Primary somatosensory cortex (S1); Ventral hippocampal commissure (vhc). Scale bar 100 µm.

**Figure 5:**
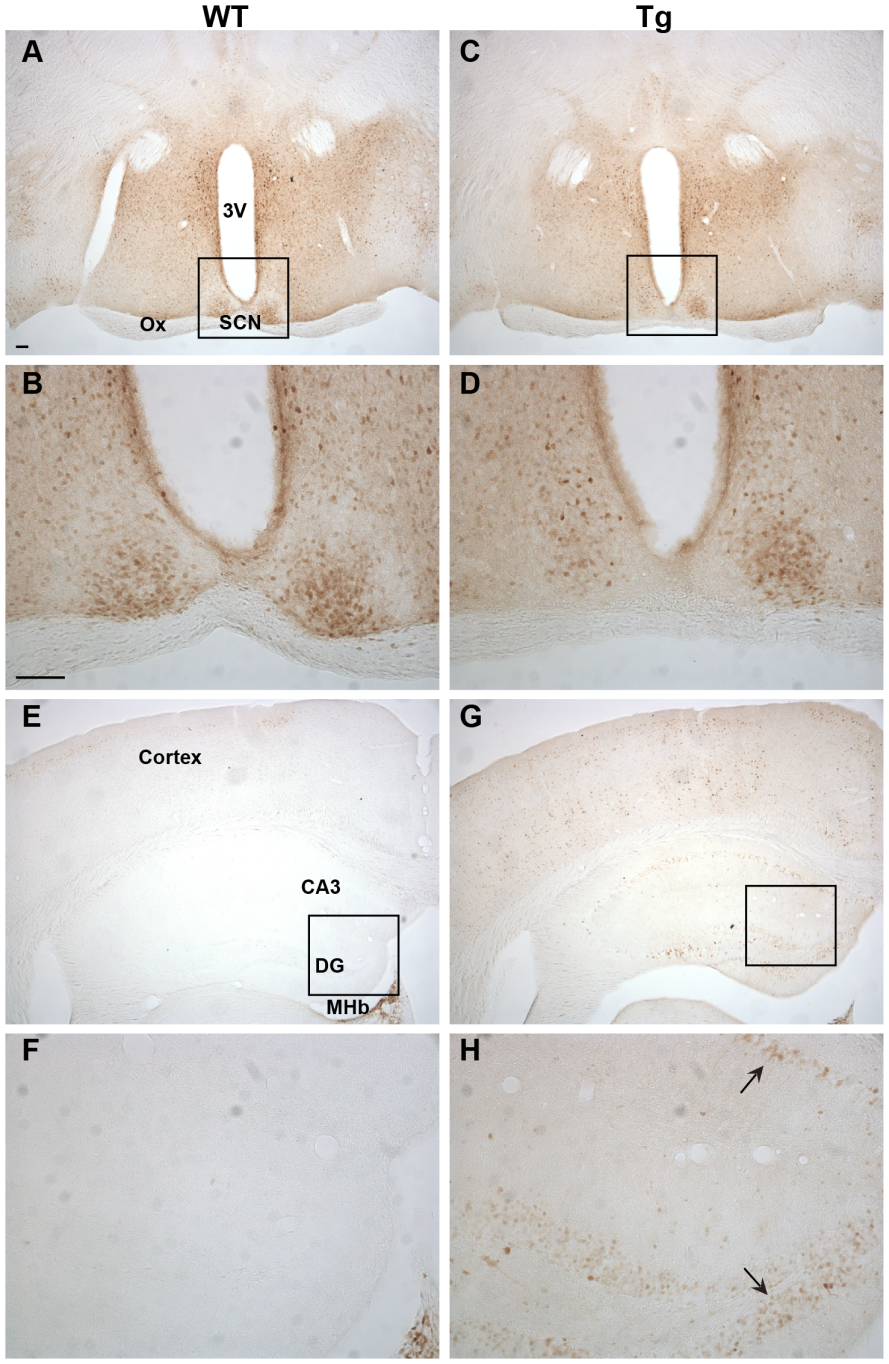
Ngb-IR in the hypothalamus and hippocampus. No difference could be seen in localization or intensity of Ngb-IR in the hypothalamus of WT (**A-B**) and Tg mice (**C-D**). The hippocampus was voided of Ngb-IR in WT mice (E-F). In Tg mice Ngb-IR was seen in cell bodies (black arrow) and processes of most structures of the hippocampus (**G-H**). Abbreviations: 3^rd^ ventricle (3V); field CA3 of hippocampus (CA3); Dentate gyrus (DG); Medial habenular nucleus (MHb); Optic chiasm (Ox). Scale bar 100 µm.

**Figure 6:**
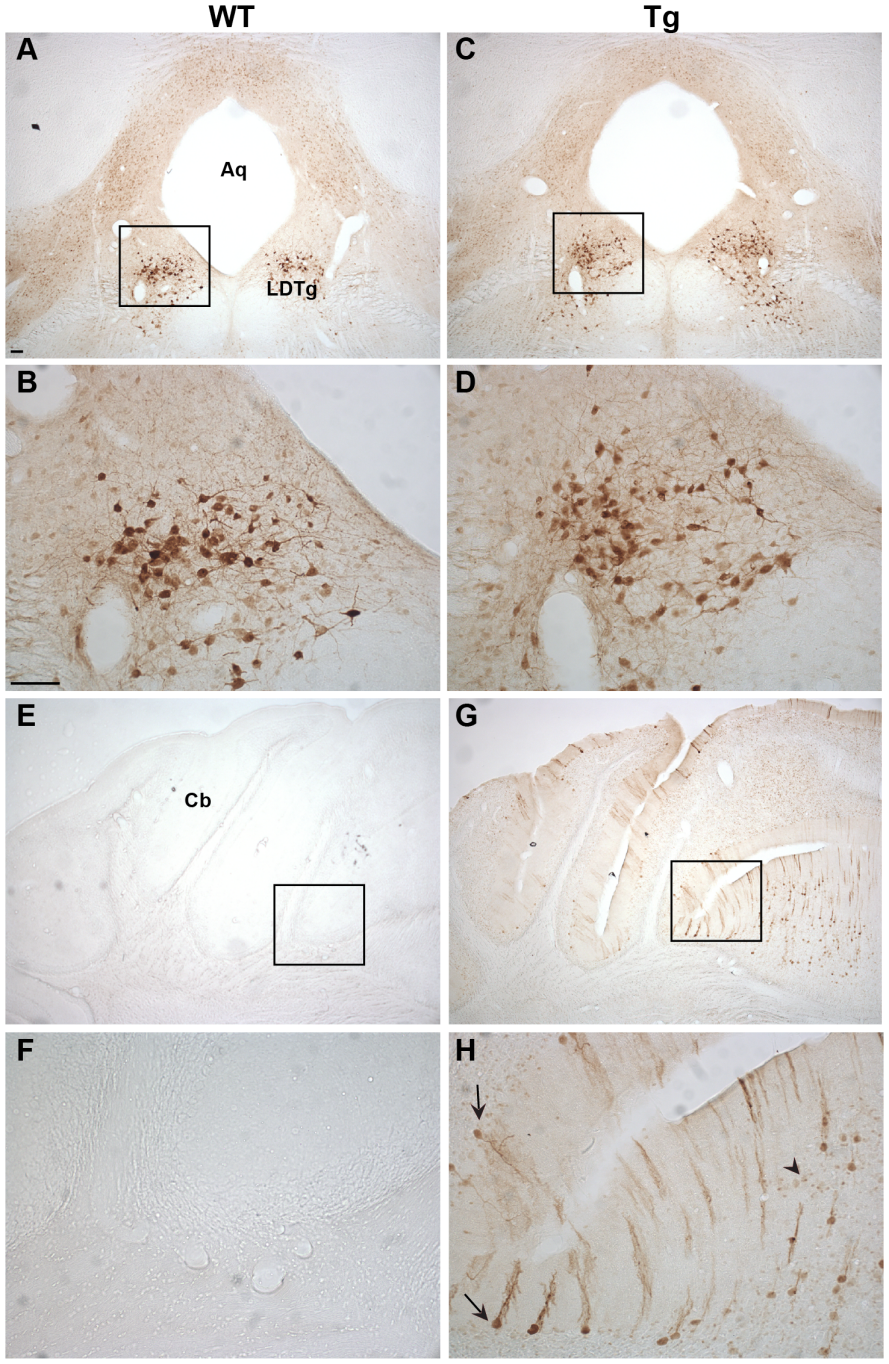
Ngb-IR in the hindbrain and cerebellum. In the hindbrain pontine nuclei no difference was seen between WT (**A-B**) and Tg (**C-D**) mice in localization, intensity or morphology of the Ngb-IR neurons. No Ngb-IR could be seen in the cerebellum of WT mice (**E-F**). In contrast, Ngb-IR was seen in Purkinje cells (black arrow) and granule cells (black arrowhead) of the cerebellar lobule in Tg mice (**G-H**). Abbreviations: Aqueduct (Aq); Cerebellum (Cb); Laterodorsal tegmental nucleus (LDTg). Scale bar 100 µm.

In the forebrain, distinct Ngb-IR was seen primarily in cortical layer II of WT mice (Figure 3A-B). Conversely, in Ngb-Tg mice Ngb-IR was seen in all cortical layers (Figure 3C-D). Ngb-IR was located in the cell soma and processes of both WT and Ngb-Tg mice. No Ngb-IR was observed in the CPu of WT mice (Figure 3E-F), whereas in Ngb-Tg mice a weak and scattered Ngb-IR could be seen (Figure 3G-H). Endogenous expression of Ngb was not affected by the transgene, as can be seen from the similarity in Ngb expression in the lateral septum (LS) between WT and Ngb-Tg mice (Figure 3E-G). The piriform cortex was one of the areas, where the differential expression was most pronounced. In WT mice, Ngb-IR was weak and restricted to a few cells (Figure 4A-B). In contrast, Ngb-Tg mice had intense Ngb-IR in both the cell soma and processes (Figure 4C-D). In the midbrain, endogenous Ngb expression was restricted to the cortical layer II (Figure 4E-F). In Ngb-Tg mice medium intense Ngb-IR was seen scattered throughout all cortical layers with most IR cells in layers II-IV and VI (Figure 4G-H). In the hypothalamus, Ngb-IR was identical in WT (Figure 5A-B) and Ngb-Tg (Figure 5C-D) mice. No Ngb-IR was observed in the hippocampus of WT mice (Figure 5E-F). In contrast, medium intense Ngb-IR was seen in the soma and processes of the cells in the fields CA1, 2 and 3 of the hippocampus (CA1-3), and the dentate gyrus (DG) (Figure 5G-H) of Ngb-Tg mice. In both WT (Figure 6A-B) and Ngb-Tg (Figure 6C-D) mice, the most intense Ngb-IR was seen in the laterodorsal tegmental nucleus (LDTg), but there was no visual difference in the cell number or intensity between the two genotypes. The cerebellum was void of Ngb-IR in WT mice (Figure 6E-F). However, Ngb-Tg mice displayed scattered medium intense Ngb-IR in the cell, soma and processes of Purkinje cells and numerous weak IR in the granule cells of the cerebellar lobule (Figure 6G-H).

### Phenotype of Ngb over expressing cells (Figures 7-8)

**Figure 7:**
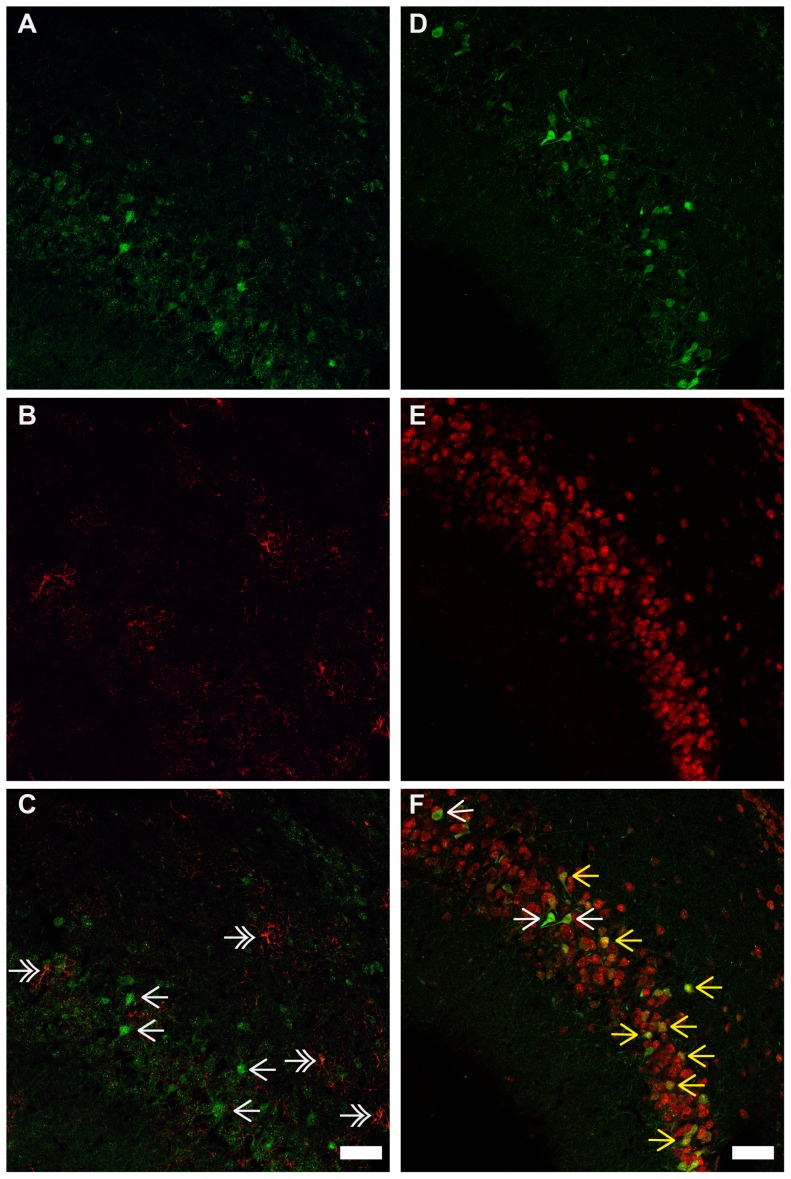
Ngb-IR, GFAP-IR and NeuN-IR in the piriform cortex. No co-localization could be observed between Ngb-IR (**A**, **C**) and GFAP-IR (**B**, **C**) in the piriform cortex. Ngb-IR (**D**, **F**) and NeuN-IR (**E**, **F**) was found to be highly co-localized in the piriform cortex. Single white arrows indicate Ngb-IR cells, double-headed white arrows exemplifies GFAP-IR cell. Yellow arrows exemplify Ngb-IR and NeuN-IR co-localized neurons. Scale bar 50 µm

**Figure 8:**
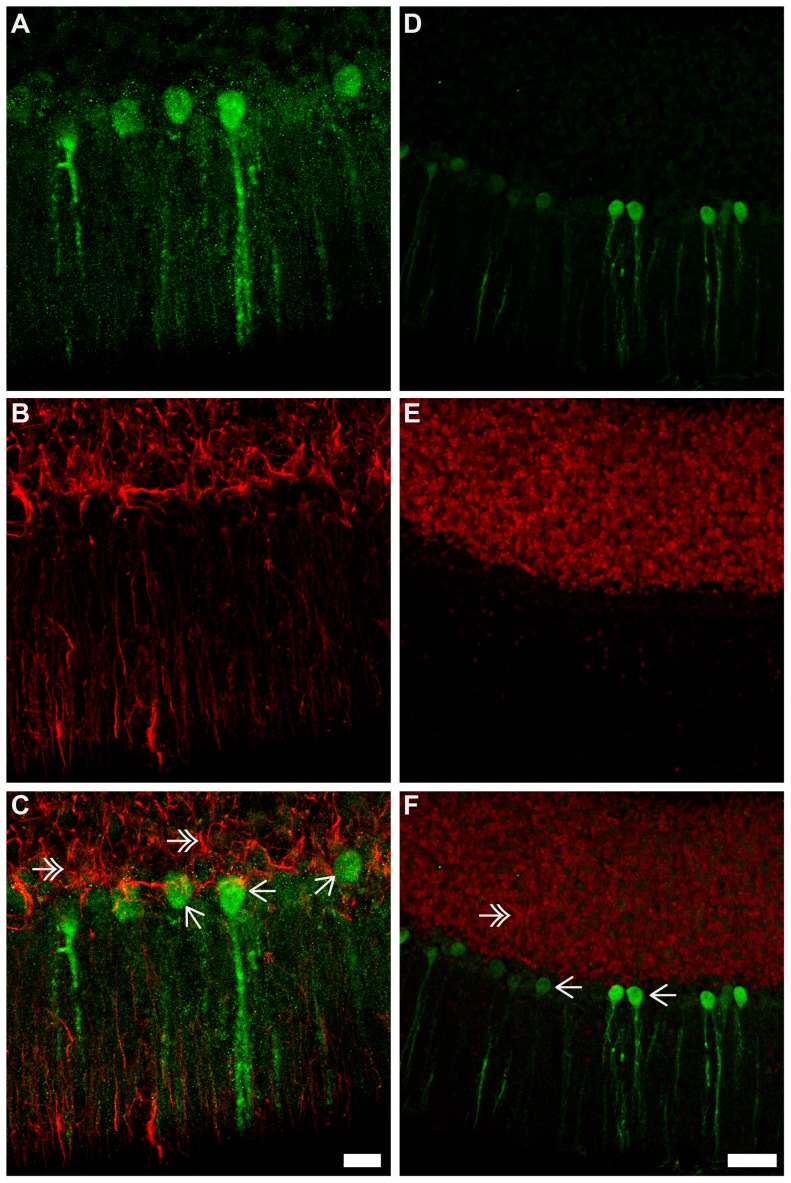
Ngb-IR, GFAP-IR and NeuN-IR in the cerebellum. In **A**-**C** Ngb (green) and the astroglia marker GFAP (red) in the cerebellum is shown. No co-localization of Ngb-IR and GFAP-IR was observed (**C**). In **D**-**E** Ngb (green) and the neuronal marker NeuN (red) is shown. The intensely Ngb stained Purkinje neurons are not co-localized with NeuN. Scale bar 20 µm in **A**-**C** and 50 µm in **D**-**E**

Antibodies specific to GFAP (astroglia) and NeuN (neuronal) were used to characterize the phenotype of the Ngb over expressing neurons in Ngb-Tg mice. No co-localization was observed between Ngb-IR and GFAP-IR throughout the brain exemplified in the piriform cortex (Figure 7G) and cerebellum (Figure 8G). In contrast Ngb-IR was seen co-localized with NeuN in the piriform cortex (Figure 7H), and in all other brain areas with positive IR. One exception was in the cerebellum, where no co-localization was seen between NeuN and Ngb-IR in Purkinje cells (Figure 8H). However, this is due to the NeuN antibody being incapable of detecting Purkinje neurons according to the information given by the manufacturer.

### Infarct volume and Ngb-IR in the ischemic penumbra of Ngb-Tg mice (Figure 9)

**Figure 9:**
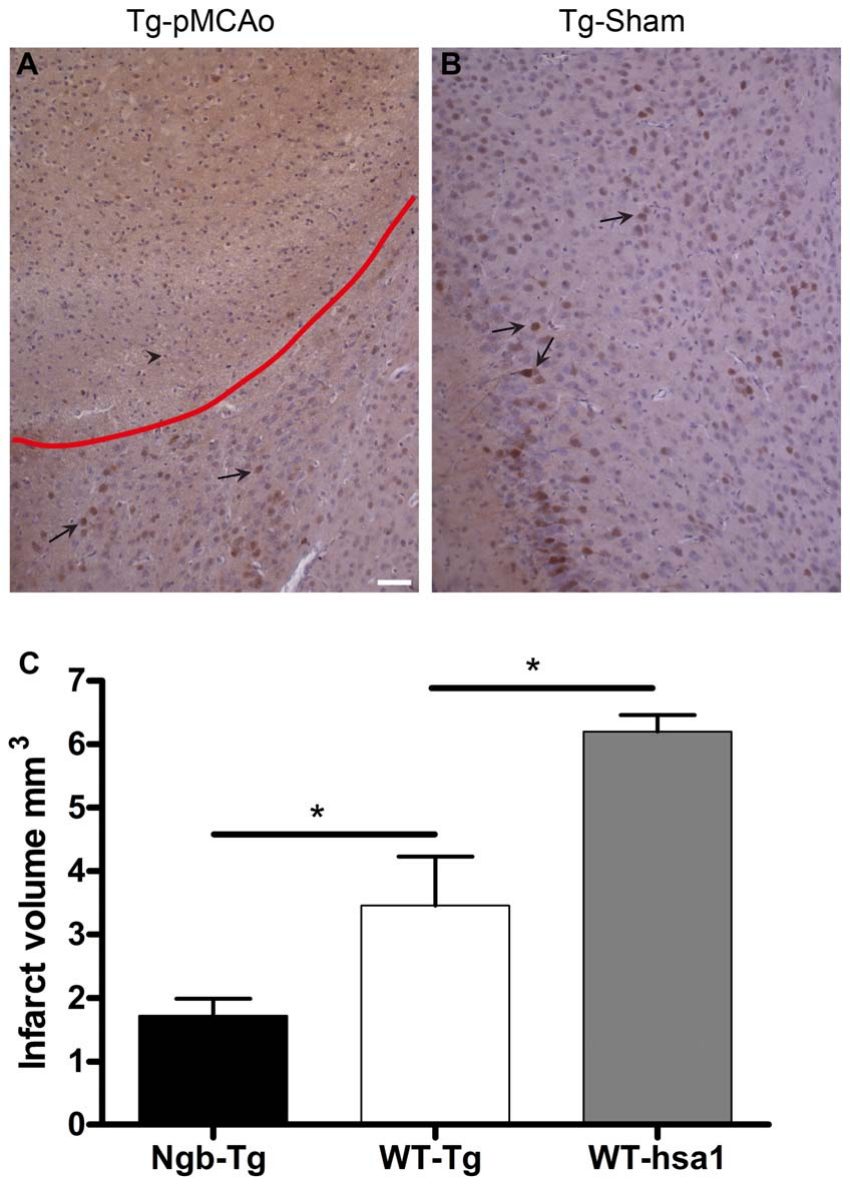
Ngb-IR in the penumbra and infarct volumes 24 hours after permanent middle cerebral artery occlusion. Ngb-IR in the cortex of a representative pMCAo operated Ngb-Tg mouse is shown in **A**. No increase in Ngb-IR intensity was observed in pMCAo operated Ngb-Tg mice when compared to sham-operated Ngb-Tg mice (**B**). The red line marks the penumbra. Black arrows exemplify Ngb-IR neurons and a black arrowhead a necrotic cell. No increase in Ngb expression is seen within nor adjacent to the penumbra area in the pMCAo operated Ngb-Tg animals compared to Tg-sham (**A**-**B**), which suggests no selective sparing of neurons expressing Ngb. Infarct volumes 24 hours after pMCAo estimated with 2D nucleator and Cavalierís principle is shown in **C**. The estimated infarct volume in cortex was significantly larger in WT Tg background mice (n = 5, total mean volume 3.5 mm^3^ SE±0.77 mm^3^) compared to Ngb-Tg (n = 7, total mean volume 1.7 mm^3^ SE±0.27 mm^3^) littermates. p<0.048. Also infarct volumes between the two different WT background strains differed significantly. The infarct volume of the WT mice with hsa1 background (n = 4 total mean volume 6.2 mm^3^ SE±0.26 mm^3^), was significantly larger compared to the infarct volume in the WT mice with Tg-background, p<0.032. Mean values are indicated by the lines and * denotes significance. Scale bar 50 µm.

Visual inspection of the cortex of three pMCAo operated Ngb-Tg mice showed no change in the number or intensity of Ngb-IR neurons in the penumbra area, compared to three sham-operated Ngb-Tg mice (Figure 9A-B). The infarct was clearly identified by pyknosis, karyohexis, karryolysis and tissue dissolution, all signs of liquefactive necrosis, which is a known type of necrosis in the brain following ischemia. No Ngb-IR neurons could be seen in the core of the infracted area, nor in the penumbra of pMCAo operated mice (Figure 9A). This suggests that Ngb positive neurons are not selectively spared under ischemic conditions compared to neurons without Ngb. Using the 2D nucleator and the Cavalieri estimator we estimated the total mean infarct volumes on Mayeŕs haematoxylin stained sections 24 hours after pMCAo. The estimated volumes differed significantly between WT mice with Tg-background (n = 5, total mean volume 3.5 mm^3^ SE±0.77 mm^3^), and Ngb-Tg littermates (n = 7, total mean volume 1.7 mm^3^ SE±0.27 mm^3^) p<0.048. Also there was a significant difference in the infarct volumes between the WT mice with Tg-background, and the WT mice with hsa1-background (n = 4, total mean volume 6.2 mm^3^ SE±0.26 mm^3^) p<0.032 (Figure 9C).

## Discussion

Several studies have shown a correlation between Ngb over expression and cell protection using transgenic mice in ischemia [19], [20], [22] and oxidative stress [38] models. In these studies, different tissue nonspecific promoters were used to drive either rodent or human Ngb expression. Also different antibodies were used to detect Ngb, making a direct one to one comparison of the degree of over expression, and the localization of Ngb, complex. In the present study, the Ngb-Tg mice developed by Greenberg and co-workers were used. In this mouse, Ngb expression is enhanced by use of a tissue nonspecific chicken β-actin promoter with a CMV enhancer, which should give a widespread distribution and a high level of Ngb over expression in the brain. In line with a previous study using adenoviral up regulation of Ngb in rats [21], these Ngb-Tg mice were shown to have significantly reduced infarct volume following tMCAo ischemia [19]. However, in the aforementioned studies, no detailed anatomical characterization of Ngb expression in the Ngb-Tg mice was given. We believe, in light of recent conflicting results concerning the *in vivo* neuroprotective effect of Ngb and the specificity of various Ngb antibodies used [26], [28], that it is important with an independent verification of the degree of over expression using thoroughly validated antibodies whose expression pattern has been compared with the staining pattern obtained when using the commercially available ones [26]. In addition, we believe an independent verification of the Ngb-Tg mice’s resistance to ischemic damage is needed.

### Model considerations: Genetics and background

The reader should take into account the underlying premise of this study is the use of a mix of hetero- and homozygote Ngb-Tg mice, which is in contrast to the study by the Greenberg group where homozygote mice were used [19]. We are aware of this potential limitation of the study design. However, this would only be a limitation if the study had been underpowered, and we were unable to detect an effect of treatment and genotype. Our power calculation showed that the study was adequately powered and the fact that a significant difference in infarct size was achieved, even in a mixed genetic background, underlines the robustness of the study design. Another potential limitation of using mixed genotypes is that the anatomical distribution of Ngb could differ between heterozygous and homozygous animals. This is probably not the case as the same expression pattern was seen in all the Ngb-Tg mice investigated with ISH and IHC. It is more likely over expressing cells will just express more Ngb in the homozygote mice, than giving rise to a vastly different expression pattern.

An important factor that can influence the results using Ngb-Tg mice from JAX© Mice instead of the original founder line is the genetic background. The original Ngb-Tg mice were founded in CD1 mice by the Greenberg group [32], but according to JAX© Mice they generated a new C57BL6 strain: “In an attempt to offer alleles on well-characterized or multiple genetic backgrounds, alleles are frequently moved to a genetic background different from that on which an allele was first characterized. It should be noted that the phenotype could vary from that originally described”. That genetic background can modify the originally observed phenotype is commonly known. The same transgene, over expressed or deleted, may produce a severe phenotype in one strain and only produce a milder one in another [39]. Therefore maintenance of strain purity of the founding mouse line during breeding is important for the background, not to add a considerable source of variability [40]. The fact that JAX© Mice frequently move alleles to different genetic backgrounds, makes the interpretation and comparison of results obtained in different laboratories complicated due to background variations.

### Level of over expression in Ngb-Tg mice

No fold change of Ngb over expression was given in the initial characterization of the Ngb-Tg mice [19], [32]. In this study, an approximately four to five fold increase in brain Ngb mRNA and protein levels were found. This is comparable to the levels reported by [22] and [20] in Ngb-Tg mouse models, where either cytomegalovirus or ubiquitin C promoters were used to up regulate human and mouse Ngb, respectively, and with a robust over expression seen using a synapsin promoter [38].

### Anatomical localization of Ngb mRNA and IR in Ngb-Tg mice

The main sites of differential Ngb mRNA and IR expression were the cortex, CPu, hippocampus and cerebellum, with the cortex and hippocampus as the areas with the most pronounced over expression. These areas were also found to over express Ngb by RT-PCR and Western blotting, but in contrast to our study, all areas were found to have about equal levels of Ngb [32]. In the rest of the brain no over expression could be seen, and the level of endogenous Ngb expression was unaffected. Taken the tissue nonspecific chicken β-actin promoter into account, the observed restricted Ngb over expression pattern is surprising. No detailed description of the Ngb expression pattern in the other Ngb-Tg models is given, although the cortex [20], hippocampus [22] and cerebrum [38] are mentioned as the areas of up regulation. From an ischemia point of view, the differential expression of Ngb in the cortex and CPu is of interest as these two areas are affected the most by the ischemic infarct. In line with Khan et al [19], an increased number of Ngb-IR cells were observed in the cortex, but only a small number of Ngb-IR cells were found in the CPu. Khan et al [19] also reported an increased expression of Ngb in astroglia, which normally do not express Ngb. This is in line with the observations made by Wang et al [20], but in contrast the study by Li et al [22] and Lee et al [38], where only neuronal expression was found. Using the neuronal marker NeuN and the astroglia marker GFAP, we show that Ngb over expression is restricted to neurons, which is at odds with the transgene promoter design. It should be noted, that a polyclonal goat anti-Ngb from Santa Cruz Biotechnology was used in the two previous studies characterizing the Ngb-Tg mice used in this study. This antibody was recently reported to give identical staining patterns in WT and Ngb-null mice indicating that it may not be suitable for IHC [26]. Antibody specificity, or lack thereof, may therefore explain some of the differences seen in Ngb expression pattern and cell-type specificity between this study and the former studies.

### Infarct volume and Ngb expression in the ischemic area of Ngb-Tg mice

In line with our previous work on Ngb expression after ischemia [24], [26] we saw no increase in Ngb-IR in the penumbra of pMCAo operated Ngb-Tg mice. This is not surprising, as ischemia is not known to increase expression of β-actin. Our data show that an elevated level of Ngb correlates with a smaller infarct volume following ischemia in Ngb-Tg mice from JAX© Mice. This study thus corroborates and expands upon the results obtained by the Greenberg group [19], as well as other studies investigating ischemic or smook induced damage in relation to Ngb over expression [20], [22], [38]. In view of the observed protective effect, it is surprising that over expression of Ngb in the infarcted area protects against development of an infarct without neurons containing Ngb, being selectively spared in the core and penumbra region. In a recent paper, we found Ngb-null mice to exhibit smaller infarct volumes following ischemia, compared to WT mice of the same background [26]. It is a paradox that expressing no Ngb at all and expressing Ngb in levels four to five fold higher than normally, confers the same phenotype in relation to reduced infarct volume following pMCAo. Khan et al [19] proposed that the observed effect of Ngb over expression was due to a direct action of Ngb within the cell. However, if Ngbs neuroprotective effect was restricted to the individual cell harboring Ngb, then based on the relative few and scattered Ngb-IR neurons in the cortex of the Tg mice, a similar pattern of islands of viable Ngb expressing cells among necrotic tissue could be expected, which we show here not to be the case. Similarly, the change in infarct size of Ngb-null mice should have been restricted to two cell layers of the cortex that would have expressed Ngb, however, the effect of Ngb deficiency was seen throughout the cortex [26]. Furthermore, a pronounced and widespread hypoxic induction of immediate early gene expression (c-FOS) in the brain of Ngb-null mice was seen in areas not normally expressing Ngb [27]. These observations indicate that the *modus operandi* of Ngb, and the ramifications of altered Ngb expression may not confined to the specific cell either over expressing or lacking Ngb. It should therefore be borne in mind that reducing or over expressing Ngb represents unphysiological states, and one must be careful when making causative conclusions about the normal function of Ngb, as compensatory mechanisms are likely to be induced and add to the phenotype [39]. It is also clear from previous studies from our group, that Ngb at normal levels does not give neurons selective salvage from ischemic or hypoxic cell death [24], [26], [27]. This indicates that the natural function of Ngb may not be related to neuroprotection *per se* and if Ngbs function really is linked to neuroprotection, then evolution would have, most likely, made sure that Ngb was present in high amounts in most brain regions. More work is needed to fully characterize the likely contribution from compensatory mechanisms to the phenotype of the transgenic Ngb mouse lines.

The final aim of the study was to evaluate the genetic background contribution to the phenotype. We compared the infarct volumes from one C57BL6 strain with the infarct volume produced in a different inbred colony originated from the same C57BL6 strain, and found a significant difference between WT C57BL6 mice with Ngb-Tg background and WT C57BL6 mice with hsa1 background. The latter mouse line was used to make Ngb-null mice. The potential magnitude of strain background is clear from a wide range of studies, showing that damage severity following experimental stroke varies among different mouse strains [41]–[44]. The organization of the cerebral arterial tree is highly variable both in size, pattern and in the extent of collateral circulation [43]. The collateral number, diameter and complexity of the arterial tree are known to be considerable contributors to damage severity following stroke in mice [43]. This difference is even apparent between mice of the same strain, but from different suppliers [45]. In fact a five-fold difference in infarct volume has been seen between studies using the same strain, same period of MCAo, as well as the same survival period [46]. In the context of the present study, it is important to recognize that the Ngb-Tg mice from JAX© Mice are hybrids of the C56BL/6, DBA/2 and CD1 mice strains. These three strains have different genetic backgrounds, and genes from all of them will be inherited in the hybrids. This could result in offspring carrying different background genes, which again may result in different phenotypes [47]. The variation in vascular anatomy seems to be a highly hereditable trait [48], and it can therefore not be excluded that the background genetics might confer different phenotypes as a result of the vascular structure of the Circle of Willi being different. At the same time, studies have shown that differences in vulnerability to ischemia may be caused by variation in neuronal and glial susceptibility, which again is inheritable [41]. The variance in the WT-Tg group was higher compared to the WT-hsa1 group, suggesting that the background in the WT-Tg group might be influenced by more variables than the WT-hsa1 group. This is a likely scenario, when considering the hybrid background of this group. However, the variance in the Ngb-Tg group was much smaller than in the comparable WT-Tg background group. This might be due to the fact that the infarct volume is smaller in the Ngb-Tg group. When the area influenced by the ischemic insult is small, any possible MCA variations, as well as other variables, will influence the result to a lesser extent. These differences in variance again suggest that there is considerable variation in the arterial tree.

## Conclusions

This is the first study to give a detailed anatomical characterization of the Ngb-Tg mouse line, commercially available at JAX© Mice. By use of thoroughly validated in-house made antibodies, increased Ngb-IR was found in the neurons of primarily the cortex, CPu, hippocampus and cerebellum. A significant reduction in infarct volume was found thus confirming the correlation between elevated Ngb levels, and neuroprotection. Lastly, we provide evidence that genetic background cause significant variation in infarct volume, and care must be taken when comparing results obtained in different mouse strains exhibiting the same transgenic expression.
